# E-classroom as a Blended Learning Tool: A Structural Equation Modelling Analysis Using Modified Technology Acceptance Model

**DOI:** 10.7759/cureus.56925

**Published:** 2024-03-25

**Authors:** Dilip Kumar, Medha Mathur, Amrita Sarkar, Meet Chauhan

**Affiliations:** 1 Community Medicine, Pacific Institute of Medical Sciences (PIMS), Udaipur, IND; 2 Community Medicine, Geetanjali Medical College and Hospital (GMCH), Udaipur, IND; 3 Community Medicine, Tomo Riba Institute of Health and Medical Sciences (TRIHMS), Naharlagun, IND; 4 Community Medicine, Shantabaa Medical College, Amreli, IND

**Keywords:** technology acceptance model, self-directed learning, medical students, medical education, google classroom, e-learning

## Abstract

Background: E-classrooms help teachers save time, keep classes organized, and improve communication with students. This study aims to assess Google Classroom's usefulness in enhancing medical students' knowledge and acceptance of new technology for in-depth learning.

Material and method: This educational interventional study was carried out on 100 students in the 3rd year of the M.B.B.S., Part 1. After a briefing about Google Classroom and educational topics, enrolled students and faculty were allowed to discuss the topic for two months. Following this, the descriptive approach was utilized to describe the respondents' technology acceptance through the administration of the technology acceptance model (TAM) survey questionnaire.

Results: Students were actively involved in discussion, with a 67% response rate. Nearly 85% of students agreed that Google Classroom is a satisfactory way for in-depth knowledge acquisition. On factor analysis, it was observed that the goodness of fit was 0.985, suggesting that the model is acceptable. It was also found that perceived usefulness (PU) had a significant positive effect on motivation towards self-directed learning (SDL), and perceived ease of use (PEOU) had a positive effect on both behavioural intention and actual use.

Conclusion: Google Classroom is a valuable tool for learning that can enhance active self-learning and increase behavioural intention and actual use. It should be incorporated into day-to-day teaching activities to overcome time constraints.

## Introduction

With the revolution of information and technology (IT), we became part of the Internet, networking, social media, etc. In this Internet era, the most affected group is the young generation. The Internet and social media can act as educational enhancers. With the proper and careful use of resource material and information from the Internet, students can gain good knowledge, study well, and achieve new heights of success.

In any education system, we generally use traditional teaching methods like chalk and board, PowerPoint presentations, demonstrations, tutorials, flip charts, audio-visual media, etc. These are one-time teaching methods. While these approaches might yield high scores for academics, incorporating complementary techniques alongside these traditional methods can offer a comprehensive understanding of a subject, thereby offering a more holistic learning experience to students who might not excel in standard assessments. The introduction of new technology into the classroom has revolutionized the teaching and learning process. The 21st-century learning environment creates exciting learning opportunities for students to collaborate and learn at their own pace, making them active participants in the learning process [1]. E-learning is a popular method of learning for young people who are proficient in digital technologies.

Newly emerging concepts in medical education, such as self-directed learning (SDL), webinars, and e-learning, are now very useful and important because they are accessible and effective. The e-classroom and programme design afforded a new paradigm for a successful online, synchronous, real-time distance learning experience conducive to the practice of identified skills and lasting knowledge [2]. Students are more likely to be able to apply what they have practiced in simulated circumstances to real life [3].

Frehywot et al. [4], in a systematic review of e-learning in medical education in resource-constrained settings, reported the major reasons for using e-learning as a faculty shortage, to cast the net wider, and to maximize the use of resources by the students. The National Medical Council (NMC) and the erstwhile Medical Council of India (MCI) are extensively using online Google groups to train medical faculty under a one-year fellowship in medical education at ten nodal centres across India. Listserv is being used as an e-learning platform in the FAIMER fellowship conducted at various centres in India.

Google Classroom is a cloud-based learning management system (LMS) that is considered one of the best platforms out there for enhancing teachers' workflow. Google Classroom helps teachers save time, keep classes organized, and improve communication with students. Google Classroom can monitor activities, keep records, and provide an easy way to assess the students without disturbing the regular teaching schedule. The purpose of this study is to investigate the attitudes and practices of medical students towards new technology and their acceptance of it. The study will use a combination of traditional teaching methods and daily exposure to Google Classroom, a web-based learning platform. This will allow the researchers to assess the students' knowledge of topics in depth and their willingness to use new technology in their learning.

## Materials and methods

Subject and settings

An educational intervention, using purposive sampling, was conducted for a short-term follow-up study of 100 third-year medical students over a period of two months from July 2018 to October 2018. Four faculty members from the community medicine department also participated in the study.

The study was conducted in two stages. In the first stage, a briefing session was held for all enrolled students and faculty members to introduce Google Classroom and the topics and activities that would be carried out. In the second stage, students and faculty members were enrolled in Google Classroom and allowed to discuss the topics in depth using videos, PowerPoint presentations, and other related materials. Students and faculty members were allowed to post materials at any time, but the active discussion time was fixed from 7 p.m. to 8 p.m. in the evening. During this time, they were given reading material related to the topic, their queries were answered, and they were given any necessary inputs. The researchers encouraged self-directed learning by asking relevant questions. This activity was carried out for two months.

The activity of enrolled students in Google Classroom was monitored using a monitoring chart with variables such as the number of times they commented, the number of duplicate comments, the frequency of resource material they provided, and how actively they discussed the topic.

Survey instrument

**Figure 1:**
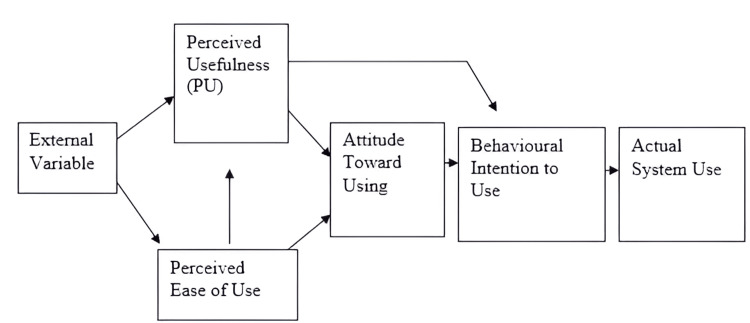
Technology acceptance model Davis, 1989 [5]

Participants received a feedback form with basic demographic information and a survey instrument known as the modified technology acceptance model (TAM) after a two-month period. The original TAM model was developed by Davis in 1989 (Figure 1) [5].

This model has 12 items that assess perceived usefulness (PU) and perceived ease of use (PEOU). The model focuses on factors that determine behavioural intention to use new technologies from the end user's perspective. It is also well established that any new technology implemented for research purposes must incorporate all three aspects of behaviour, attitude, and practice. In this study, we adopted an instrument from the study of Al-Maroof and Al-Emran [6], which included two additional aspects: intention to use (IT) and actual system use (AU). We also added an instrument called "Google Classroom Motivated for SDL" to assess the impact of Google Classroom on learning skills. All responses were recorded on a scale of 4 (strongly agree) to 1 (strongly disagree).

Statistical analysis

The internal consistency of the data was assessed using two measures: Cronbach's alpha coefficient and McDonald's omega. Cronbach's alpha is a measure of how well the items on a scale measure the same construct. McDonald's omega is a more robust measure of internal consistency that is less sensitive to the number of items on a scale. According to Hair et al. [7], a loading of 0.7 or higher is considered reliable for each item in a factor analysis. Before conducting factor analysis, we first assessed the factorability of the data matrix by calculating the Kaiser-Meyer-Olkin (KMO) coefficient and performing the Bartlett sphericity test. The KMO coefficient is a measure of sampling adequacy, which indicates how well the data are suited for factor analysis. The Bartlett sphericity test is a statistical test that is used to determine whether the correlation matrix is an identity matrix. If the value of the KMO coefficient is greater than 0.85 and the p-value for Bartlett’s test of sphericity is less than 0.001, then factor analysis can be performed. Second, a principal components analysis (PCA) with varimax rotation was carried out on the 18 items of the questionnaire. Third, confirmatory factor analysis (CFA) was performed. The goodness of fit of the model was evaluated with different indices such as chi-square, comparative fit index (CFI), Tucker-Lewis’s index (TLI), and root mean square error of approximation (RMSEA). Values of CFI and TLI between 0.90 and 0.95 were considered acceptable for the goodness of fit of a model, while values greater than 0.95 revealed excellent goodness of fit. Hu and Bentler suggested that a value of less than 0.05 for RMSEA is considered acceptable [8]. Third, the study employed structural equation modeling (SEM) to test the hypothesized relationships between the variables. SEM is a statistical technique that is used to test causal relationships between latent variables. Latent variables are constructs that cannot be directly observed but are inferred from observed variables. Smart PLS is a software package that is used to conduct SEM analyses.

Hypothesis

PU and PEOU have been identified as key factors in the acceptance and use of new technology in a number of studies [9-13]. SEM consists of latent and observed variables connected by paths and can solve multi-regression problems and factor analysis between the constructs that were determined based on theory [14].

With respect to this context, this study tests the following hypothesis. H1: the PU of Google Classroom influences the user’s motivation towards the SDL; H2: the PEOU of Google Classroom has an influence on the user’s motivation towards the SDL; H3: the IT of Google Classroom has an influence on the user’s motivation towards the SDL; H4: the PU influences the PEOU of Google Classroom; H5: PEOU influences the IT of Google Classroom; H6: IT influences the AU of Google Classroom; H7: AU influences the SDL of Google Classroom; H8: PEOU influences the AU of Google Classroom; and H9: PU influences the AU of Google Classroom.

We also included a few feedback questions related to the participants' experience with the topic discussed in Google Classroom and the effectiveness of Google Classroom for gaining in-depth knowledge of the topic.

Data analysis

The collected data were entered in MS Office Excel 2013 format (Microsoft® Corp., Redmond, WA). All statistical analyses were conducted using the statistical software Jamovi 2.3.26 and Smart PLS version 3 [15]. The results were presented in the form of tables and graphs. Proportions were used to summarize categorical data, and the mean and standard deviation (SD) were calculated for TAM factors.

Ethical consideration

Prior ethical clearance was taken from institutional ethics committee.

## Results

This study investigated the impact of Google Classroom on student motivation towards SDL and behavioural intention. A total of 398 responses were received from 100 students, 315 of which were in writing form and 83 in various material forms (Table 1).

**Table 1:** Number of responses in the form of comments and material shared by various participants

Participants	Responses	Material shared
Student (n=100)	315	83
Faculty member (n=4)	158	34
Total	473	117

The results showed that about 85% of students agreed that Google Classroom is a satisfactory way for in-depth knowledge acquisition and will continue using it in the future (Table 2).

**Table 2:** Distribution of participants according to feedback

Feedback questions	Strongly disagree	Disagree	Agree	Strongly agree
Satisfactory experience with Google Classroom	9.4	15.5	31.3	43.8
Appropriateness and quality of material	3.0	9.4	37.6	50.0
Contribution to in-depth knowledge	3.1	11.2	41.9	43.8
Continuation in future	0.0	10.4	42.7	46.9

In order to measure the reliability of each item, factor loading was measured. In the present study, the overall alpha value was 0.79, and McDonald’s omega value was 0.8, which indicated the reliability of the scale item. Thus, the constructs’ convergent validity is established (Table 3).

**Table 3:** Central tendency of individual items of Modified TAM scale and overall reliability of scale construct (Item Reliability Statistics) IT: intention to use, PU: perceived usefulness, PEOU: perceived ease of usefulness, AU: actual system use, SD: standard deviation

Factor	Items		Mean	SD	Cronbach's alpha of factor
	Overall scale reliability		2.53	0.51	0.79
PU	A	Enhances my efficiency	3.8	1.12	0.910
	B	Enhances my learning productivity	3.64	1.01	
	C	Enables me to accomplish tasks more quickly	3.54	1.30	
	D	Improves my performance	3.76	1.18	
	E	Saves my time	3.84	1.19	
	F	Does not have any distinctive features	3.61	1.27	
	G	Not applicable to all academic courses	3.72	1.08	
PEOU	H	Easy to use	3.6	1.15	0.820
	I	Enables me to access the course materials	3.68	0.85	
	J	Convenient and user-friendly	3.72	1.33	
	K	Allows me to submit my assignments	3.42	0.92	
	L	Requires no training	2.98	1.19	
	M	Makes it easier to avoid future academic difficulties	3.4	0.82	
IT	N	I intend to increase my use of the Google Classroom	2.6	1.11	0.876
	O	It is worth to recommend the Google Classroom for other students	3.1	1.09	
	P	I’m interested to use the Google Classroom more frequently in the future	2.8	1.17	
AU	Q	I use the Google Classroom on daily basis.	2.78	1.11	0.862
	R	I use the Google Classroom frequently.	3.02	1.12	

A KMO value above 0.6 and a highly statistically significant Bartlett's test of sphericity (<0.001) confirm the data's adequacy. Later, a principal component analysis using varimax rotation was executed, and a total of three factors emerged, which accounted for 57.2% of the total variance. Confirmatory factor analysis derived from the factor model shows CFI = 0.984 and RMSEA = 0.032. Overall, the goodness of fit was 0.985, suggesting that the model is acceptable (Table 4).

**Table 4:** Kaiser-Meyer-Olkin, Bartlett's test, percent of variance explained by factors and goodness-of-fit indices KMO: Kaiser-Meyer-Olkin, RMSEA: root mean square error of approximation, GFI: Goodness of Fit Index, PGFI: Parsimony Goodness of Fit Index, MFI: McDonald Fit Index, CFI: Comparative Fit Index

Component of factor analysis	Values
KMO (measure of sampling adequacy)	0.827
Bartlett's test of sphericity	Chi-square: 795
	df: 120
	Sig. <0.001
Factor 1	26.5 (% of variance)
Factor 2	17.0 (% of variance)
Factor 3	13.7 (% of variance)
Total variance explained	57.2%
Goodness of fit indices	Values
χ²	126
P-value	<0.001
RMSEA	0.032
GFI	0.985
PGFI	0.718
MFI	0.736
CFI	0.984

**Figure 2:**
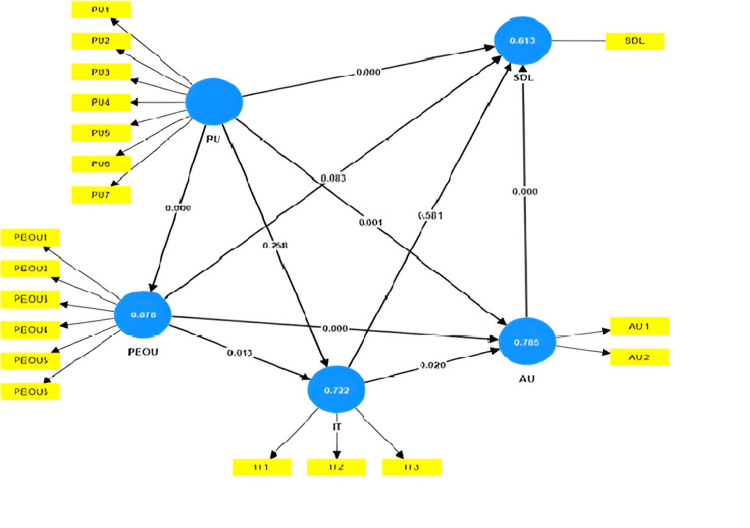
Structural equation model SDL: self-directed learning; IT: intention to use; PU: perceived usefulness; PEOU: perceived ease of usefulness; AU: actual system use

The path analysis results supported hypotheses H1, H4, H5, H6, H7, and H8, but did not support hypotheses H2, H3, and H9. This means that PU had a significant effect on motivation towards SDL, and PEOU had a positive effect on behavioural intention and actual use (Table 5 and Figure 2).

**Table 5:** Path coefficient - standard deviation, T-value, and p-value *Significant p-value (<0.05) SDL: self-directed learning; IT: intention to use, PU: perceived usefulness, PEOU: perceived ease of usefulness, AU: actual system use

Path	Standard deviation	T statistics	P-values	Hypotheses
AU -> SDL	0.151	4.079	0.000*	Supported
IT -> AU	0.109	2.326	0.020*	Supported
IT -> SDL	0.165	0.552	0.581	Not supported
PEOU -> AU	0.146	4.95	0.000*	Supported
PEOU -> IT	0.241	2.492	0.013*	Supported
PEOU -> SDL	0.318	1.734	0.083	Not supported
PU -> AU	0.118	3.267	0.001*	Supported
PU -> IT	0.234	1.131	0.258	Not supported
PU -> PEOU	0.016	60.177	0.000*	Supported
PU -> SDL	0.189	4.378	0.000*	Supported

## Discussion

In the third professional year, Part 1, 100 students participated in this educational interventional short-term follow-up study.

It was observed that 85% of students agreed that Google Classroom is a satisfactory way for in-depth knowledge acquisition and will continue using it in the future. According to a study by Nanthinii, 50% of students agreed that they would be more active in learning through Google Classroom and that it had significantly improved their interest in learning English [16]. A study by Hussaini et al. found that Google Classroom is effective in improving students' access to learning, attentiveness towards learning, and knowledge acquisition through self-study [17].

In the present study, 87% of students agreed that the material and knowledge provided in Google Classroom were appropriate. Shaharanee et al. found that about 80% of respondents strongly agreed that lecturers were friendly, approachable, and easily contacted in Google Classroom [18].

In the present study, we found that the overall alpha value was 0.79 and the McDonald's omega value was 0.8. These values indicate that the scale items are reliable, meaning that they measure the same construct consistently. The individual scale items also had Cronbach's alpha values of 0.7 or above. This further supports the reliability of the scale items. Thus, the constructs’ convergent validity is established. The Cronbach’s alpha and composite reliability values are greater than 0.7, and the AVE values are greater than 0.5 [6].

In the present study, the mean score for perceived usefulness and ease of use was 3.7 and 3.47, respectively. This means that most students found Google Classroom to be useful and easy to use. The mean scores for intention to use and actual use were 2.83 and 2.9, respectively. This means that while most students intended to use Google Classroom, they actually used it less frequently. A similar study reported that perceived usefulness, ease of use, and behavioural intention (BI) to use were all high. However, actual system use was lower [19]. A similar study conducted in Oman also showed that all variables were substantially successful in terms of both behavioural intention and actual Google Classroom use [6]. In a study by Iftakhar, it was observed that low internet speed was a hindrance to actual system use [20].

In the present study, the goodness of fit was 0.985, suggesting that the model is acceptable. A similar finding was observed in the study by Pratama [13].

The results of SEM in this study suggest that PU has a significant effect on motivation towards SDL, and PEOU and PU both have a significant effect on AU. It was also seen that AU enhances motivation for SDL. Various studies have found that both PEOU and PU positively affect the behavioural intentions of those who perceive the use of Google Classroom as easy, useful, and an enhancer for self-directed learning [6,13,18,21]. Kalayou et al. found that perceived usefulness has a significant influence on attitude and intention to use, and perceived ease of use has a significant influence on perceived usefulness and intention to use [9]. According to Albashtawi and Al Bataineh, the usefulness of Google Classroom was ranked first, and ease of use was ranked second [22]. According to Fauzi et al., PEOU has a significant positive effect on PU because the benefits of Google Classroom affect students' attitudes, such as comfort and pleasure, which are highly experienced when greater levels of usefulness are felt during learning [14]. Another study showed that the attitude towards using (ATU) variable has a positive and significant effect on the BI, the BI variable has a positive and significant effect on the AU variable, and the PEOU variable has a positive and significant effect on the PU variable [23].

## Conclusions

The use of technology in teaching and learning has been found to enhance active self-learning. Therefore, it should be incorporated into day-to-day teaching activities. The shortfall in time duration may also be overcome if the topics are taught in Google Classroom along with traditional teaching. This study suggests that Google Classroom is a valuable tool for learning and that students find it effective in helping them acquire knowledge. The study found that Google Classroom can be a motivating tool for SDL and can increase behavioural intention and actual use. However, further research is needed to investigate the impact of other factors, such as social influence and facilitating conditions, on the use of Google Classroom. This study was conducted with a small sample size, so the findings may not be generalizable to larger populations. Future studies should be conducted with larger populations to improve the external validity and generalizability of the findings. Another limitation of online learning is that some students may initially be active but gradually become less active. This emphasizes the importance of self-motivation for active learning.

## Appendices

**Table 6:** Survey instrument PU: perceived usefulness, PEOU: perceived ease of use, BI: behavioural intention to use, AU: actual system use

Variables	Score: Strongly disagree - 1, Disagree - 2, Neutral - 3, Agree - 4, Strongly agree - 5
PU	
PU1: Google Classroom enhances my efficiency.	
PU2: Google Classroom enhances my learning productivity.	
PU3: Google Classroom enables me to accomplish tasks more quickly.	
PU4: Google Classroom improves my performance.	
PU5: Google Classroom saves my time.	
PU6: Google Classroom doesn’t have any distinctive useful features.	
PU7: Google Classroom is not applicable to all academic courses.	
PEOU	
PE1: Google Classroom is easy to use.	
PE2: Google Classroom enables me to access the course material.	
PE3: Google Classroom is convenient and user-friendly.	
PE4: Google Classroom allows me to submit my assignments.	
PE5: Google Classroom requires no training.	
PE6: Google Classroom makes it easier to avoid future academic difficulties.	
BI	
BI1: I intend to increase my use of the Google Classroom.	
BI2: It is worth to recommend the Google Classroom for other students.	
BI3: I’m interested to use the Google Classroom more frequently in the future.	
AU	
AU1: I use the Google Classroom on daily basis.	
AU2: I use the Google Classroom frequently.	
